# High karyotypic variation in *Orthemis* Hagen, 1861 species, with insights about the neo-XY in *Orthemisambinigra* Calvert, 1909 (Libellulidae, Odonata)

**DOI:** 10.3897/CompCytogen.v15.i4.68761

**Published:** 2021-11-03

**Authors:** Liliana M. Mola, María Florencia Fourastié, Silvia Susana Agopian

**Affiliations:** 1 Laboratorio de Citogenética y Evolución - Departamento de Ecología, Genética y Evolución, Instituto de Ecología, Genética y Evolución (CONICET-UBA), Facultad de Ciencias Exactas y Naturales, Universidad de Buenos Aires, Buenos Aires, Argentina; 2 Instituto de Ecología, Genética y Evolución (CONICET-UBA), Facultad de Ciencias Exactas y Naturales, Universidad de Buenos Aires, Buenos Aires, Argentina; 3 Scientist working privately, CABA, Argentina

**Keywords:** Chromosomal evolution, holokinetic chromosomes, heterochromatin characterization, sex-determination system

## Abstract

The American dragonfly genus *Orthemis* Hagen, 1861 is mainly found in the Neotropical region. Seven of 28 taxonomically described species have been reported from Argentina. Chromosome studies performed on this genus showed a wide variation in chromosome number and a high frequency of the neoXY chromosomal sex-determination system, although the sexual pair was not observed in all cases. This work analyzes the spermatogenesis of *Orthemisdiscolor* (Burmeister, 1839), *O.nodiplaga* Karsch, 1891 and *O.ambinigra* Calvert, 1909 in individuals from the provinces of Misiones and Buenos Aires, Argentina. *Orthemisdiscolor* has 2n=23, n=11+X and one larger bivalent. *Orthemisnodiplaga* exhibits the largest chromosome number of the order, 2n=41, n=20+X and small chromosomes. *Orthemisambinigra* shows a reduced complement, 2n=12, n=5+neo-XY, large-sized chromosomes, and a homomorphic sex bivalent. Fusions and fragmentations are the main evolutionary mechanisms in Odonata, as well as in other organisms with holokinetic chromosomes. *Orthemisnodiplaga* would have originated by nine autosomal fragmentations from the ancestral karyotype of the genus (2n=22A+X in males). We argue that the diploid number 23 in *Orthemis* has a secondary origin from the ancestral karyotype of family Libellulidae (2n=25). The complement of *O.ambinigra* would have arisen from five autosomal fusions and the insertion of the X chromosome into a fused autosome. C-banding and DAPI/CMA_3_ staining allowed the identification of the sexual bivalent, which revealed the presence of constitutive heterochromatin. We propose that the chromosome with intermediate C-staining intensity and three medial heterochromatic regions corresponds to the neo-Y and that the neo-system of this species has an ancient evolutionary origin. Moreover, we discuss on the mechanisms involved in the karyotypic evolution of this genus, the characteristics of the neo sex-determining systems and the patterns of heterochromatin distribution, quantity and base pair richness.

## Introduction

Family Libellulidae is characterized by having a modal number 2n=25 (n=12+X) in males, an XX/X0 chromosomal sex-determination system and chromosomes that decrease gradually in size, with the X chromosome being one of the smaller in the complement (Mola et al. 1999; Mola 2007; Kuznetsova and Golub 2020). *Orthemis* Hagen, 1861 is a common genus of American dragonflies mainly located in the Neotropical region. Seven of the 28 taxonomically described species have been reported from Argentina (*Orthemisaequilibris* Calvert, 1909, *Orthemisambinigra* Calvert, 1909, *Orthemisambirufa* Calvert, 1909, *Orthemiscultriformis* Calvert, 1899, *Orthemisdiscolor* (Burmeister, 1839), *Orthemisnodiplaga* Karsch, 1891 and *Orthemisphillipi* von Ellenrieder, 2009) (von Ellenrieder 2012; Mauffray and Tennessen 2019; Lozano et al. 2020).

Chromosome studies performed on this genus have revealed two particular characteristics: first, a wide variation in chromosome number, ranging from 2n=7 with two bivalents and a trivalent in meiosis (n=2II+1III) in *Orthemislevis* Calvert, 1906 to 2n=41 (n=20+X0) in *O.nodiplaga*, with no species having the characteristic modal number 25 of Libellulidae; and, second, a high frequency of neoXYsex-determining systems (Cumming 1964; Cruden 1968; Kiauta and Boyes 1972; Ferreira et al. 1979; Kiauta 1979; Souza Bueno 1982; Agopian and Mola 1984a). Taking into account the small size of the X chromosome in Odonata, the recognition of a heteromorphic sex bivalent at the different meiotic stages is difficult, as it depends on both the size of the autosome with which it was fused, and the degree of contraction of the bivalents. About half of the species in the order with this neoXY system have a homomorphic sex bivalent in males, including the species of *Orthemis* (Oksala 1943; Cumming 1964; Kiauta 1971, 1972, 1979; Ferreira et al. 1979; Souza Bueno 1982; Mola 1996; Perepelov et al. 1998; Perepelov and Bugrov 2002).

In Odonata, as in most organisms with holokinetic chromosomes, karyotype evolution might have occurred through fusions and fragmentations. Both types of rearrangements are favored because no limitations are imposed by the centromere (Kiauta 1969b; Mola and Papeschi 2006; Mola 2007; Kuznetsova and Golub 2020).

The heterochromatin is one of the key components of the genome and its biology is based on both the repetitive DNA sequences and the proteins specifically bound to this DNA. Although many of the structural and functional characteristics of heterochromatin remain to be elucidated, there is evidence that its content and distribution affect DNA replication, modulate chromosome structure, and play a role in karyotypic evolution, gene expression and differentiation, and in genome organization and evolution (Hennig 1999; Redi et al. 2001; Straub 2003). The C-banding staining technique is frequently used to detect heterochromatin, allowing the visualization of most of the constitutive heterochromatin segments. The use of base-specific fluorochromes improves the characterization of heterochromatic regions with regard to their relative enrichment with AT or GC base pairs. The most widely used fluorochromes are CMA_3_ and DAPI, which preferentially stain GC- and AT-rich DNA zones, respectively.

The heterochromatin in monocentric chromosomes is mainly located in centromeric and nucleolar organizer regions (NORs), while in holokinetic chromosomes it is predominantly located in the telomeric regions, with variations in base pair richness and distribution among different holokinetic systems (Mola and Papeschi 2006). Almost 80 species of Odonata have been studied with C-banding, of which 75% belong to seven families of Anisoptera and the rest to six families of Zygoptera. Libellulidae includes the greatest number of species analyzed (about 33% of the total).

C-banding revealed that, in general, autosomes present heterochromatic blocks in both telomeric regions. These blocks are small or large, symmetric or asymmetric. The free sex chromosome of males is entirely C-positive, shows intermediate staining, or has C-positive bands only located in terminal or interstitial regions (Suzuki and Saitoh 1988; Perepelov et al. 1998, 2001; Nokkala et al. 2002; Perepelov and Bugrov 2002; Walia and Chahal 2014; Kuznetsova et al. 2018; Walia and Devi 2018). In about half of the species so far analyzed, the X chromosome is entirely C-positive, which is consistent with its allocycly (facultative heterochromatinization) during male meiosis and reflects a different degree of condensation rather than the presence of constitutive chromatin.

In terms of base pair richness, it may be AT-rich or GC-rich, with variations between species, chromosomes of the same species and even within the same chromosome (Grozeva and Marinov 2007; De Gennaro et al. 2008; Walia and Katnoria 2017; Walia and Chahal 2018, 2019, 2020; Walia et al. 2018a, 2018b; Walia and Somal 2019; Walia and Devi 2020).

Taking into account the broad karyotypic variation observed within *Orthemis* and aiming to elucidate the mechanisms involved in the karyotypic evolution of this genus, our study analyzes the meiotic development, karyotype and patterns of heterochromatin distribution, quantity and base pair richness in *Orthemisdiscolor*, *O.ambinigra* and *O.nodiplaga*. In addition, we identify the homomorphic neo-XY sex pair of *O.ambinigra* with C-banding and fluorescent staining and propose a hypothesis of its origin.

## Methods

The present study was performed on nine adult males of *Orthemisdiscolor*: three males from Santo Pipó (27°08'28"S, 55°24'32"W), five males from Parque Nacional Iguazú (25°41'35"S, 54°26'12"W) and one male from María Magdalena (26°14'15"S, 54°36'13"W) (Misiones Province), three adult males of *O.nodiplaga* from Parque Pereyra Iraola (34°50'38"S, 58°08'56"W) (Buenos Aires Province) and 19 adult males of *O.ambinigra*: 14 males from Delta del Paraná (34°25'15"S, 58°32'31"W) (Buenos Aires Province) and five males from Parque Nacional Iguazú (25°41'35"S, 54°26'12"W) (Misiones Province), Argentina. Administración de Parques Nacionales, Argentina, issued the permit for collection and transport of material from the Parque Nacional Iguazú.

The specimens were etherized in the field, a dorsal longitudinal cut in the abdomen was made and they were whole fixed in 3:1 (absolute ethanol: glacial acetic acid). Later, the gonads were dissected out and placed in fresh fixative for one day and stored in 70% ethanol at 4 °C. For meiotic studies a piece of gonad was placed in 45% acetic acid for 2 or 3 min to facilitate cell spreading and slides were made by the squash method in iron propionic hematoxylin.

C-Banding, fluorescent staining with CMA_3_ (chromomycin A_3_) and DAPI (4’-6-diamidino-2-phenylindole) and Feulgen staining were carried out on unstained slides. A piece of gonad was squashed in 45% acetic acid, the coverslip was removed by the dry-ice method and the slide was air-dried. For C-banding, slides were first dehydrated in absolute ethanol, followed by hydrolysis with 0.2N HCL at 60 °C for 30–60 sec., then, they were treated with a saturated solution of Ba(OH)_2_ at room temperature for 15–20 min., incubated in 2XSSC at 60 °C for 1 h, stained with 2% Giemsa in Phosphate Buffer at pH 6.8, washed in tap water, air-dried and mounted (Giraldez et al. 1979). The sequential DAPI-CMA_3_ banding was performed using the technique described by Rebagliati et al. (2003). For Feulgen staining, slides were washed twice in distilled water for 10 min each, and then air-dried and hydrolyzed in 5 N HCl at 25 °C for 60 min. This was followed by washing twice in distilled water for 10 min each, air-drying, staining with Schiff’s reagent for 2 h in the dark, washing twice in SO_2_ water for 10 min each, air-drying, washing twice in distilled water for 5 min each, air-drying and mounting.

## Results

**Figure 1.**
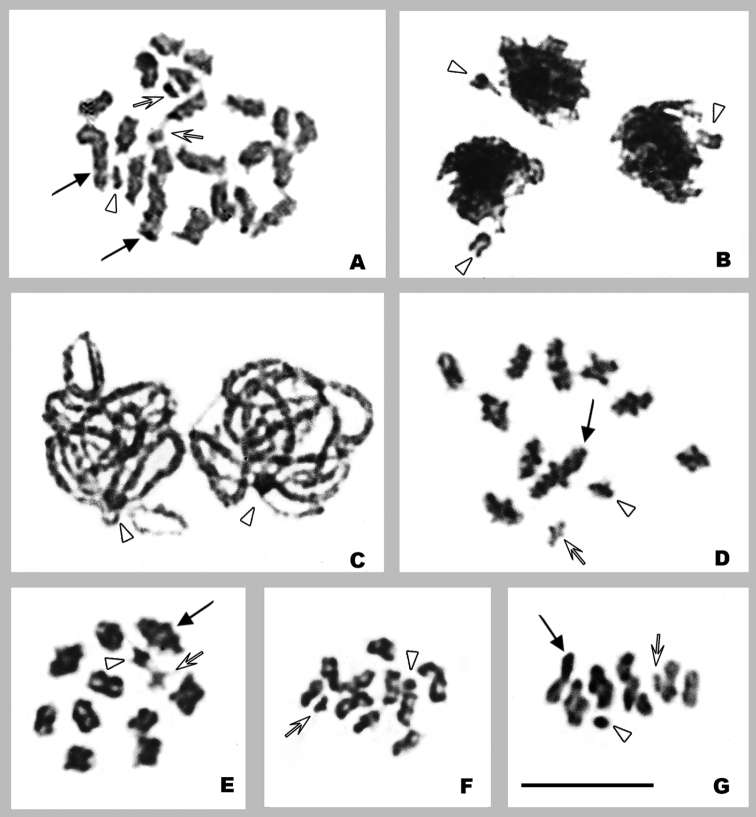
*Orthemisdiscolor***A** spermatogonial prometaphase **B** zygotene **C** pachytene **D** diakinesis **E** prometaphase I **F** prophase II **G** metaphase II. Arrowheads point X chromosome. White arrows point *m* chromosomes. Black arrows point larger pair. Scale bar: 10 µm.

*Orthemisdiscolor* presents 2n=23, n=11+X, with no chromosomal differences between locations. At spermatogonial prometaphase the X chromosome, a pair of small chromosomes (*m* chromosomes), and one larger pair are distinguished (Fig. 1A). At both spermatogonial prometaphase and metaphase, the chromosomes always show thin associations between some telomeric regions.

At early prophase I, the X chromosome is isopycnotic or slightly negatively heteropycnotic and is separated from the chromatin mass formed by the autosomes (Fig. 1B). At pachytene, the X chromosome is isopycnotic or slightly positively heteropycnotic and is located near to the nuclear periphery, associated with the telomeric regions of some autosomes (Fig. 1C). At diakinesis and prometaphase I, bivalents present one subterminal chiasma. At this stage, a large bivalent and the *m* bivalent – of similar size to that of the X chromosome – are distinguished, while the rest of the bivalents decrease gradually in size (Fig. 1D, E). The X chromosome divides equationally at anaphase I and there are 12 chromosomes in all cells during the second division. At prophase II, the autosomes adopt a ε shape (Fig. 1F) and at metaphase II, the X chromosome is somewhat separated from the autosomes on the equatorial plate (Fig. 1G).

*Orthemisnodiplaga* presents 2n=41, n=20+X (Agopian and Mola 1984a). The study of new specimens allowed us to confirm previous results and describe the meiotic stages. At spermatogonial metaphase the chromosomes vary in size, and the X chromosome is distinguished because it is the largest of the complement (See Fig. 2 in Agopian and Mola 1984a).

**Figure 2.**
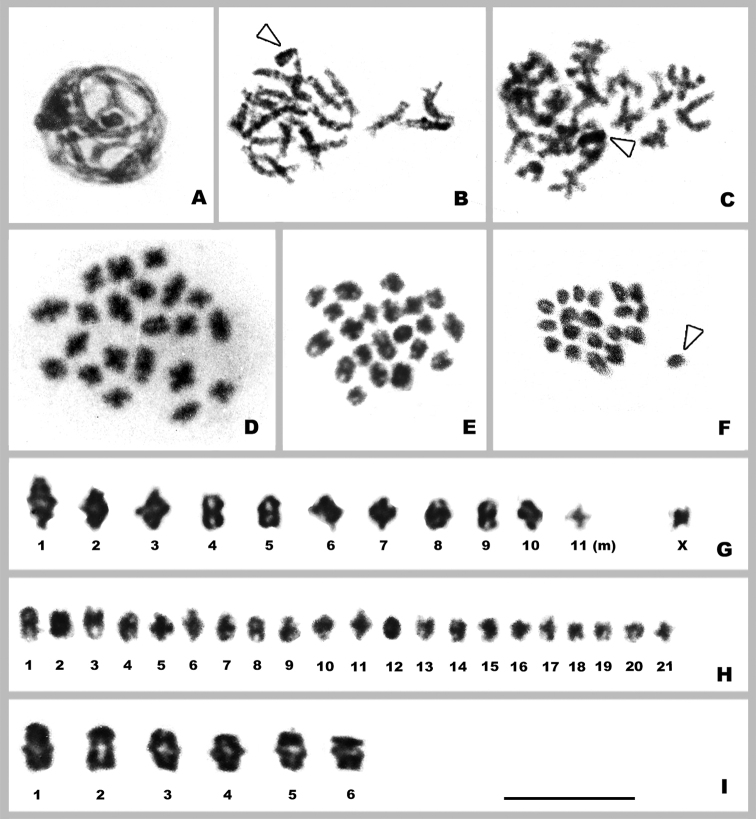
*Orthemisnodiplaga* (**A–F**) and meiotic karyotypes of *Orthemis* species (**G–I**) **A** pachytene **B** late pachytene **C** diplotene **D** diakinesis **E** prometaphase I **F** metaphase II **G***O.discolor* (from Fig. 1E) **H***O.nodiplaga* (from Fig. 2E) **I***O.ambinigra* (from Fig. 3H). Arrowheads point X chromosomes. Scale bar: 10 µm.

At pachytene, the X chromosome is isopycnotic or slightly positively heteropycnotic and at late pachytene, the bivalents show separate telomeric zones (Fig. 2A, B). From diplotene onward, the bivalents exhibit one subterminal chiasma, and less often, one medial chiasma (Fig. 2C). At diakinesis and prometaphase I, the bivalents decrease slightly in size, no *m* bivalent is distinguished and the X chromosome is of similar size to that of medium bivalents (Fig. 2D, E). All the metaphases II exhibit 21 chromosomes and the X chromosome is separated from the autosomes (Fig. 2F). The bivalents of this species are smaller than those of *O.discolor* (Fig. 2G, H).

**Figure 3.**
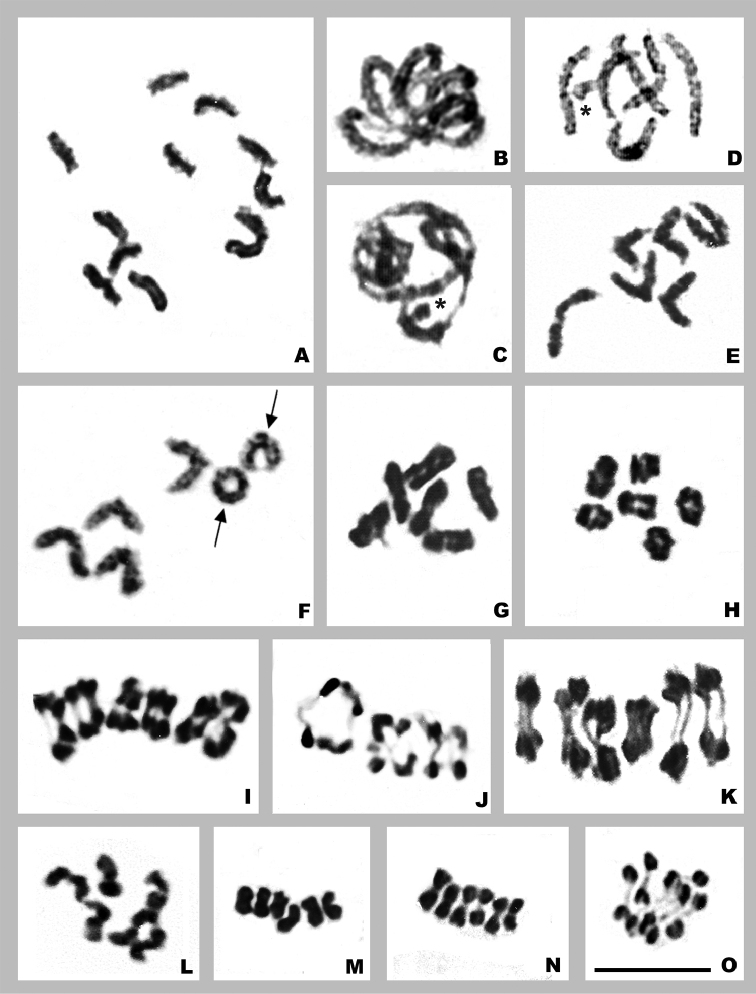
*Orthemisambinigra***A** spermatogonial prometaphase **B** bouquet stage **C** pachytene **D** late pachytene **E** diplotene **F** early diakinesis with one bivalent with two terminal chiasmata **G** diakinesise **H** prometaphase I **I** early anaphase I **J** early anaphase I (Feulgen staining) **K** medium anaphase I **L** prophase II **M** prometaphase II **N** early anaphase II **O** anaphase II. Asterisks point interstitial loop. Black arrows point bivalent with two chiasmata. Scale bar: 10 µm.

*Orthemisambinigra* presents 2n=12 and n=5+neo-XY, with no chromosomal differences between individuals from distinct geographical locations. At spermatogonial prometaphase, the chromosomes are of similar size (Fig. 3A).

At early pachytene, no positive heteropycnotic bodies are observed and bivalents are often arranged in a bouquet (Fig. 3B). At mid and late pachytene, one bivalent shows a small interstitial loop (Fig. 3C, D). At diplotene and diakinesis, the six bivalents are homomorphic and present one subterminal chiasma, and one or unusually two bivalents seldom show two terminal chiasmata (Fig. 3E–G). At prometaphase I, two slightly larger bivalents are identified, and none of the six bivalents are heteromorphic (Fig. 3H). The bivalents of this species are comparable to the largest bivalent of *O.discolor* (Fig. 2G, I). The low number of bivalents and long duration of anaphase I (as indicated by the large number of cells at this stage) allowed us to perform a detailed analysis. At early anaphase, chromatids that migrate to the same pole lie approximately parallel to the equatorial plane, with the two medial telomeric regions held together and moving a little ahead. The two outer telomeric regions are connected by thin chromatin threads to the outer telomeric regions that migrate to the other pole, and exceptionally, there may be thin chromatin threads between the medial telomeric regions (Fig. 3I). The DNA presence in the threads was confirmed with Feulgen staining (Fig. 3J). These chromatin threads remain until late telophase (Fig. 3K). There is no true interkinesis because chromosomes do not undergo complete despiralization. At prophase II, the chromatids remain joined by the same telomeric regions as at anaphase I and adopt the characteristic ε shape (Fig. 3L). At prometaphase II, the free telomeres of the chromatids with ε shape get close to the central ones, which remain associated with each other and adopt an 8 shape (Fig. 3M). Later chromatids rotate so that U-shaped chromosomes face the poles at metaphase II and persist in this arrangement in anaphase II, where thin chromatin threads attached to chromosome ends moving poleward are observed again (Fig. 3N, O).

### Heterochromatin characterization

*Orthemisdiscolor* exhibits C-positive bands in the telomeric region of all bivalents, either symmetric or asymmetric, in the scarce pachytenes and diakinesis able to be analyzed (data not shown).

**Figure 4.**
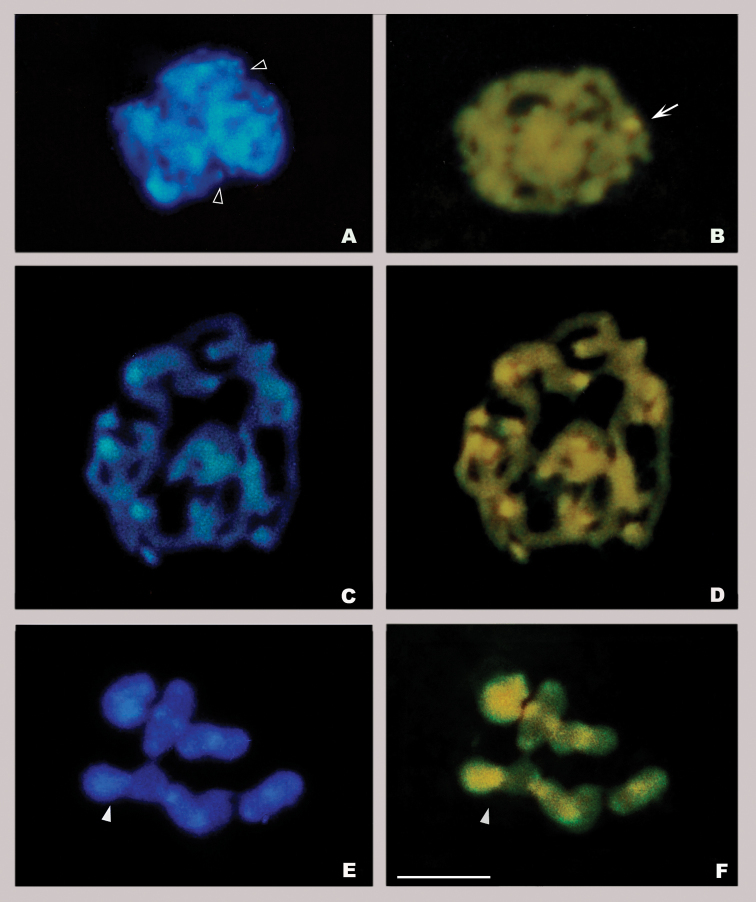
DAPI/CMA_3_ staining of *Orthemisnodiplaga* (**A, B**) and *Orthemisambinigra* (**C–F**) **A, B** zygotene **C, D** pachytene **E, F** diakinesis. Arrow points to DAPI dull/CMA_3_ bright band. Empty arrowheads point to small DAPI bright bands. White arrowheads point to neo-XY bivalent. Scale bar: 10 µm.

*Orthemisnodiplaga* has small DAPI bright bands in the telomeric region of all the chromosomes except in the telomeric region of a pair of chromosomes that shows one DAPI dull/CMA_3_ bright band at zygotene-pachytene (DAPI dull band not shown) (Fig. 4A, B).

In *O.ambinigra* the fluorescent banding indicates that from pachytene onward, the telomeric regions of all bivalents present DAPI bright/CMA_3_ bright bands (Fig. 4C–F). At diakinesis, a heteromorphic bivalent is observed; one of the homologues shows a DAPI bright/CMA_3_ bright staining, which is slightly less intense than that in the telomeric region (Fig. 4E, F).

**Figure 5.**
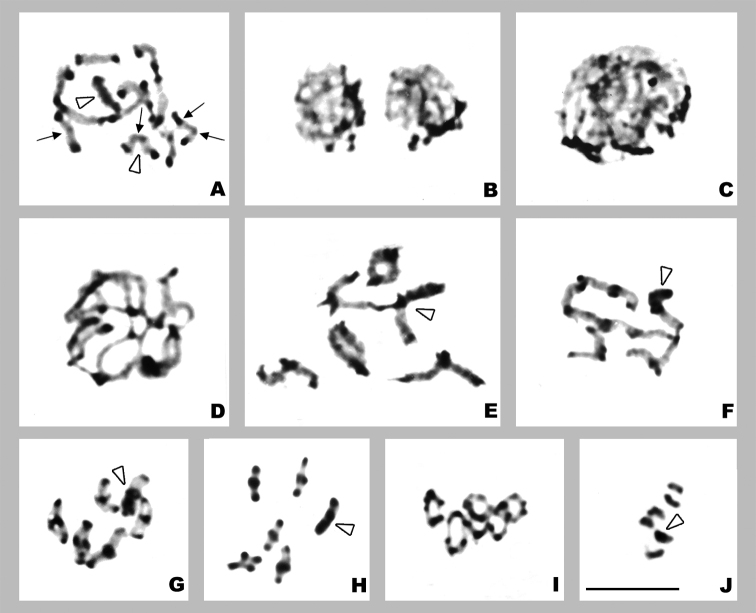
C-Banding of *Orthemisambinigra***A** spermatogonial prometaphase **B** zygotene **C** pachytene **D** early diplotene **E, F** diplotene **G** diakinesis **H** prometaphase I **I** early anaphase I **J** metaphase II. Arrowheads point to neo-XY bivalent/chromosomes. Black arrows point subterminal and interstitial bands. Scale bar: 10 µm.

In *Orthemisambinigra*, all chromosomes at spermatogonial prometaphase show C-positive bands in the telomeric region and a few chromosomes have a C-positive band adjacent to this region; some chromosomes also exhibit a C-positive bands in their interstitial region, which are less stained than those in the telomeric region, and one chromosome exhibit two interstitial bands slightly separated. In addition, one chromosome shows intermediate C-staining intensity along its length, where two or three darker regions can be distinguished (Fig. 5A). Also at this stage, several chromosomes show nonspecific associations between telomeric C bands. At zygotene, the C-positive regions show polarization at the nuclear periphery and this bouquet arrangement persists through pachytene (Fig. 5B, C). During diplotene, bivalents show a single C-band in the telomeric region and there are associations between the bands of different chromosomes (Fig. 5D–F). From mid-diplotene onward, one of the larger bivalents is heteromorphic, one homologue has C-positive bands in the telomeric region and the other one is almost completely C-positive. In the latter, at this stage, staining allows visualizing three large interstitial and closely located C-bands, besides the telomeric bands (Fig. 5E, F). From diakinesis onward, these three bands are unified into one band due to chromosome condensation (Fig. 5G, H). At anaphase I each chromosome presents four C-bands; the central ones are very close to each other and the outer ones are connected to those of the chromosome moving to the opposite pole by thin chromatin threads (Fig. 5I). At metaphase II, the chromosomes present terminal C-bands and the chromatid of one chromosome is entirely C-positive (Fig. 5J).

## Discussion

### Karyotype evolution in the genus *Orthemis*

None of the species studied showed the modal chromosome number of the family Libellulidae (2n=25, n=12+X in males), while the complement 2n=23/24, n=11+X0/11+XX (male/female) is present in 50% of the species (Table 1). Kiauta and Boyes (1972) assumed that the typical number of the genus would be of primary origin, as opposed of being derived from the modal number of the family. However, the presence of a larger bivalent in some species (see below) suggests that this complement derived from the typical complement of the family and originated in the common ancestor of the genus by an autosomal fusion.

**Table 1.** Chromosomal data of *Orthemis* species.

Specie	2n	n	N	Locality	References
*Orthemislevis* Calvert, 1906	7	2 II+1III	2	Arround Buena Vista, Santa Cruz Department, Bolivia	Cumming 1964
*Orthemis* sp.^†^	10	4+neo-XY	4	Near Buena Vista, Santa Cruz Department, Bolivia	Cumming 1964 as *O.ferrugínea*
*O.aequilibris* Calvert, 1909	12	5+neo-XY	1	Borro-Borro, Paramaaribo District, Surinam	Kiauta 1979
*O.ambinigra* Calvert, 1909	12	5+neo-XY	14	Delta del Paraná, Buenos Aires Province, Argentina	Agopian and Mola 1984b,
			5	Parque Nacional Iguazú, Misiones Province, Argentina	Mola 2007, this work
*O.biolleyi* Calvert, 1906	23	11+X0	–	Eastern Bolivia	Cumming 1964
*O.cultriformis* Calvert, 1899	23	11+X0	–	Eastern Bolivia	Cumming 1964
			3	Cruzeiro do Sul, Acre State, Brazil	Ferreira et al 1979
*O.discolor* (Burmeister, 1839)		11+X0	1	Zanderij, Para District, Surinam	Kiauta 1979 as *O.ferruginea*
	23	11+X0^†^	4	Zanderij, Para District, Surinam	
	(25)	(11+neo-XY)^†^			
		(10+neo-XY)^†^			
	23	11+X0	1	Cieneguilla, Lima Province, Perú	Kiauta and Boyes 1972 as *O.ferruginea*
	24 F	–	1		
		11+X0	2	Rio Claro, São Paulo State, Brazil	Ferreira et al. 1979 as *O.ferruginea*
		11+X0	2	Borecéia, São Paulo State, Brazil	Souza Bueno 1982 as *O.ferruginea*
		11+X0	1	Florianopolis, Santa Catarina State, Brazil	
	23	11+X0	3	Santo Pipó, Misiones Province, Argentina	Mola 2007, this work,
	23	11+X0	5	Parque Nacional Iguazú, Misiones Province, Argentina	This work
	23	11+X0	1	María Magdalena, Misiones Province, Argentina	This work
*O.ferruginea* (Fabricius, 1775)	23	11+X0	–	Central Texas State, US	Cumming 1964
			–	Marshall Co., Oklahoma State, US	Cruden 1968
*O.ferruginea* or *O.discolor*^‡^	23	11+X0	–	Tikal, Peten Department, Guatemala	Cruden 1968
Unnamed Antillean form sp.^‡^	23	11+X0	–	Commonwealth Dominica	Cruden 1968
*O.nodiplaga* Karsch, 1891	41	20+X0	2	Parque Peryra Iraola, Buenos Aires Province, Argentina	Agopian and Mola 1988
			1	Buenos Aires City, Argentina	
			3	Parque Peryra Iraola, Buenos Aires Province, Argentina	This work

**Notes**: N: Number of individuals analyzed; F: female; † See Discussion; ‡ According to Donnelly (1995) and Paulson (1998).

In the genus *Orthemis* there is a tendency towards reduction in chromosome number through fusions. *Orthemisnodiplaga* seems to be the exception, in which the karyotype derived from nine autosomal fragmentations within the ancestral karyotype of the genus that became fixed, while two pairs of autosomes were not fragmented. It is worthwhile to mention that the X chromosome remained intact despite the high number of fragmentations, and turned out to be the largest chromosome of the complement of this species (Agopian and Mola 1984a; this work).

The modal chromosome number is present in five species (Table 1). *Orthemisdiscolor* and *O.ferruginea* (Fabricius, 1775) represent an extremely similar, closely related sibling species pair occurring in sympatry from southern US to Costa Rica (Donnelly 1995; Paulson 1998). This raises questions about the identity of the species from Guatemala analyzed by Cruden (1968) and also if the species described by Cumming (1964) from Central Texas is *O.ferruginea* (Table 1).

*Orthemisdiscolor* is the most cytogenetically studied species. Kiauta (1979) reported two morphs from Surinam, one with the modal complement and the other with variations in the chromosome number and in the sex-determining system (Table 1). In the latter, the neo-XY found in 25% of the cells is indistinguishable. The fact that the author expressed the results as a percentage of cells from all four specimens prevents us from knowing if some individuals had neo-XY and others X0 or if both types of sex chromosomes were present in all the individuals, though the latter assumption seems much less likely.

*Orthemisdiscolor* and *O.cultriformis* exhibit a distinguishable largest pair (Cumming 1964; Kiauta and Boyes 1972; Ferreira et al. 1979; Kiauta 1979; Souza Bueno 1982; this work). Cumming (1964) and Cruden (1968) do not provide a detailed analysis of the autosomal karyotype for *O.ferruginea*, *O.biolleyi* Calvert, 1906 and the unnamed Antillean form species.

The four remaining species show a markedly reduced complement, 2n=7 in *Orthemislevis*, 2n=10 in *Orthemis* sp. and 2n=12 in *O.aequilibris* and *O.ambinigra*, originated by fusions between autosomes or an autosome and the X chromosome (Table 1). The individuals from Bolivia with 2n=10 described by Cumming (1964) as *O.ferruginea* do not correspond to this species. *Orthemisferruginea* is found in North America and occur in Central America as far as to Costa Rica (Donnelly 1995; Paulson 1998).

We propose that the complement of *O.ambinigra* originated from the ancestral karyotype of the genus (2n=22+X) by five fusions of non-homologous autosomes in pairs, which eventually became fixed in the population, and by the interstitial insertion of the X chromosome in one of them leading to the neo-XY system (see below). The autosomal fusions occurred between chromosomes of different size, giving rise to a karyotype with chromosomes of similar size, where the largest autosomal pair of the ancestral karyotype remained unchanged. All or a large proportion of the telomeric heterochromatin would have been lost in the course of the multiple fusions that originated this complement. This is reflected in the current karyotype by the absence of interstitial heterochromatin in the chromosomes; in the few cases where the interstitial heterochromatin is present, it is less conspicuous than that in the telomeric regions.

### Heterochromatin distribution, quantity and base pair richness

In Odonata, heterochromatin characterization has been mainly carried out using C-banding and in a less extent with DAPI/CMA_3_ staining. Most species present heterochromatic blocks in both telomeric regions of the autosomes (Frankovič and Jurečič 1989; Prasad and Thomas 1992; Perepelov et al. 1998, 2001; Perepelov and Bugrov 2001, 2002; Nokkala et al. 2002; Grozeva and Marinov 2007; Mola 2007; Walia et al. 2011, 2016a, 2018a, 2018b; Walia and Chahal 2014, 2018, 2019, 2020; Walia and Katnoria 2017, 2018; Kuznetsova et al. 2018, 2020; Walia and Devi 2018, 2020; Walia and Somal 2019; this work). There are exceptions, where some or all the chromosomes of the complement show blocks in only one telomeric region or some chromosomes lack heterochromatin (Prasad and Thomas 1992; Perepelov et al. 2001; Walia et al. 2016b; Walia and Katnoria 2017; Kuznetsova et al. 2018, 2020; Walia and Devi 2018). The amount quantity of heterochromatin may vary from large blocks to tiny ones. In turn, these blocks may be symmetric (of equal size in both telomeric regions) or asymmetric (Perepelov et al. 1998). Species having a large amount quantity of heterochromatin show natural banding similar to C-banding in telomeric heterochromatin zones, as is the case of *Brachymesiafurcata* (Hagen, 1861) (Agopian and Mola 1988). The presence of distinct heterochromatic blocks in all autosomes is the most common feature, which is also observed in the three especies of *Orthemis* studied herein.

A few species possess interstitial or subtelomeric blocks. The subtelomeric blocks, distinguished at pachytene or spermatogonial prometaphase, are usually small and are seen in some or all of the bivalents. As chromosome condensation proceeds, these blocks fuse with those in the telomeric region into a single block (Perepelov et al. 1998, 2001; Nokkala et al. 2002; Kuznetsova et al. 2018, 2020). The interstitial blocks are found in one or a few chromosomes and are detected until late prophase I (Perepelov et al. 1998; Walia et al 2016a, 2016b; Kuznetsova et al. 2018; Walia and Katnoria 2018). *Orthemisambinigra* has both subtelomeric and interstitial blocks, the latter would be remnants of the multiple fusions that originated its reduced chromosome complement.

In Odonata, base-specific fluorochromes (DAPI/CMA_3_) have been scarcely used for heterochromatin characterization. *Somatochloraborisi* Marinov, 2001 presents bright bands in the telomeric region of most bivalents with variable base pair richness, each one being AT- or GC-rich even in the same chromosome (Grozeva and Marinov 2007). In some Anisoptera and Zygoptera species collected from India, the heterochromatin in the terminal bands show variation in base pair richness. They are mainly AT- and GC-rich or only AT-rich (Walia and Katnoria 2017; Walia and Chahal 2018, 2019, 2020; Walia et al. 2018a, 2018b; Walia and Somal 2019; Walia and Devi 2020). *Orthemisambinigra*, herein studied, also shows large bands of telomeric heterochromatin with interspersed AT- and GC-rich blocks (equally localized DAPI and CMA_3_ bright bands). *Orthemisnodiplaga*, as well as *Erythrodiplaxnigricans* (Rambur, 1842), has small heterochromatic AT-rich bands in the telomeric region of all chromosomes, except for one GC-rich band in a telomeric region of one pair of chromosomes (De Gennaro et al. 2008; this work). *Coryphaeschnaperrensi* (McLachlan, 1887) shows only one GC-rich band in the telomeric region of the largest pair, in this pair a correlation was established between the nucleolar organiser region (NOR) and the GC-rich band (De Gennaro et al. 2008). The association between GC-rich bands and NORs is frequent in insects with holokinetic chromosomes (Mola 2007; De Gennaro et al. 2008). The GC-rich telomeric band of *O.nodiplaga* and *E.nigricans* might correspond to the nucleolus organizer region as well.

Despite the small number of studies using fluorescent staining, our results support the hypothesis that the telomeric heterochromatin of Odonata has a heterogeneous base pair richness (Mola 2007).

### Characterization and origin of the neo-XY in Orthemisambinigra

In Odonata, the recognition of a heteromorphic sex bivalent in all meiotic stages is difficult, and about half of the species with this neo sex chromosomes system have a homomorphic sex bivalent in males. Its presence is inferred by the even number of chromosomes in the spermatogonial cells, the absence of a univalent in the first meiotic division and the absence of a chromosome that migrates ahead in the second meiotic division, which is a characteristic behavior of the free X chromosome in most of the species studied. In *Aeshnagrandis* (Linnaeus, 1758), for instance, the sex bivalent is heteromorphic in both the first and second meiotic divisions, but in *Erythrodiplaxmedia* Borror, 1942 the heteromorphism of the sex bivalent is recognized only at diplotene and diakinesis because it is masked by strong chromosome contraction at metaphase I (Oksala 1943; Kiauta 1969a; Mola 1996; Perepelov and Bugrov 2002).

The heterochromatin characterization of the neoXY has only been performed in three species of *Aeschna* Fabricius, 1775 (Perepelov et al. 1998; Perepelov and Bugrov 2002). Perepelov and Bugrov (2002) conclude that the formation of the neo-XY/neo-XX system in three *Aeschna* species was accompanied by the heterochromatinization of the autosomal regions of the neo-X and neo-Y, though these chromosomes are partially heterochromatic.

Several authors hypothesized that the evolution of the sex chromosomes of different insect orders such as orthopterans, lepidopterans and dipterans included total or partial loss of recombination, inactivation or loss of genes and progressive accumulation of repetitive DNAs and heterochromatinization of the Y (or W) or neo-Y chromosomes (Fuková et al. 2005; Vitková et al. 2007; Kaiser and Bachtrog 2010; Castillo et al. 2010a, 2010b, 2014; Bidau et al. 2011; Sahara et al. 2012; Zhou et al 2013; Palacios-Gimenez et al. 2015, 2018; Jetybayev et al. 2017; Buleu et al. 2020). The first step in Y chromosome degeneration is determined by the accumulation of transposable elements and their enrichment along a degenerating Y chromosome could explain the shift from euchromatic to heterochromatic chromatin structure (Steinemann and Steinemann 1998; Charlesworth et al. 2005).

**Figure 6.**
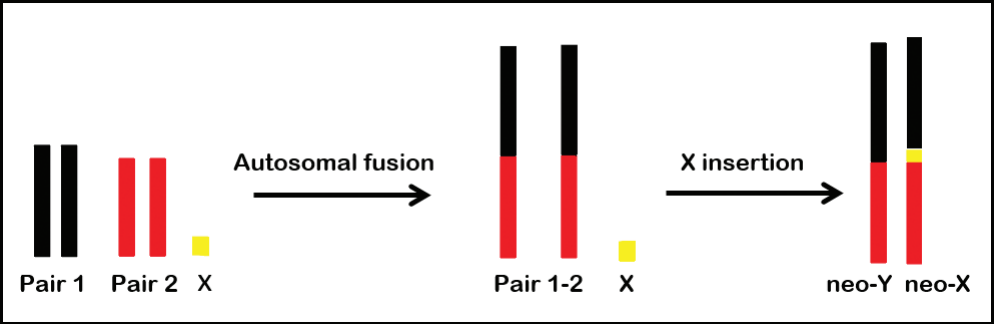
Diagram of the chromosome rearrangements that could give rise to the neo-X and neo-Y chromosomes of *Orthemisambinigra*.

Taking this into account, we propose that in *O.ambinigra* the chromosome mostly C-positive, with three large interstitial regions and two telomeric C-bands should correspond to the neo-Y chromosome. Besides, we propose that the bivalent that at pachytene presents a submedial loop should be the neo-XY. This loop may correspond to the original X chromosome of the neo-X, with no homology in the neo-Y. Likewise, the chromosome at mitosis with two interstitial C-positive bands slightly separated and telomeric C-bands should also correspond to the neo-X. These submedial bands could delimit the site of insertion of the original X into one of the fused autosomes, thus indicating that the X telomeric heterochromatin was not completely lost due to insertion (Fig. 6). We propose that the sex chromosomes should correspond to a fused pair due to its large size. An alternative hypothesis of the origin of this neo-system could be that the fusion of the X chromosome with an autosome (forming a neo-X chromosome) occurred first, and this was followed by the fusion of both members of another autosomal pair to the neo-X and neo-Y. The first hypothesis appears to be the most plausible, providing the most parsimonious explanation for the origin of the neo-system. Since the fusion of the two homologous with other two chromosomes (neo-X, neo-Y) in the same orientation is less evolutionary probable than the insertion of the X chromosome in a fused chromosome, which had previously become structural homozygous by crossing. In contrast to the results reported by Perepelov and Bugrov (2002) for the species of *Aeschna*, in our study *O.ambinigra* showed no evidence of heterochromatinization of the autosomal region of the neo-X.

Given that in *O.ambinigra* the bivalents present a single subterminal chiasma, the differentiation of the neo-Y from the homologous autosomal region of the neo-X would be facilitated by the accumulation of repetitive DNA sequences, which can modify chromatin structure leading to its heterochromatinization. On this basis, the presence of three interstitial blocks of heterochromatin in the neo-Y may indicate an advanced evolutionary stage of a neo-XX/neo-XY sex determination system in this species.
